# Physical Education

**Published:** 1889-09

**Authors:** C. F. M’Guire

**Affiliations:** Brooklyn, N. Y.


### SELECTIONS.

### Physical Education.

BY C. F. M'GUIRE, M. D., BROOKLYN, N. Y.

At present the subject of Physical Education is engaging the attention of educators to an extent that is surprising. When we consider its importance, however, we are more surprised at its almost universal neglect hitherto. The ancients paid great attention to this department of education, and, moreover, did not consider physical strength incompatible with intellectual vigor; on the contrary, their idea of the subject was aptly expressed by the axiom: "a sound mind in a sound body."

It is strange that this important fact was lost sight of by modern educators, and that we should only now be attempting what was an accomplished fact hundreds of years ago. We have had physical training, but usually mercenary in intent, and not in connection with a regular course of study. Yet people have always admired the trained athlete, and no doubt would have taken up the subject, if they knew how to go about the matter. The trouble has arisen, in a great measure, from the fact that the men who were accepted as the exponents of this art, were not as a rule men whom fathers of families would care to have their children associated with.

Comparatively a short time ago, a body of educated men took the matter up, and, as a consequence, we are beginning now to see in our schools and colleges the fruits of their laudable efforts. It is for this reason that Dr. Anderson, of the Brooklyn Normal Training School for Physical Culture, has been trying for some time past to get the medical profession interested in this matter, especially the younger members, who have more time at their disposal, and to whom it offers a lucrative business. Dr. Anderson reasons that this department of education rightly belongs to the medical profession, as its members are the only ones that are qualified, both by training and education, to fulfil properly the functions of a teacher of this art.

I would suggest that doctors, before engaging in this profession, should master some one of the various systems now practised, as by so doing they will have a foundation to start from and also save themselves much trouble and anxiety. After they have acquired the necessary ground-work, they can then begin originating, improving, or correcting, as they may find necessary.

There is a great deal to be accomplished in this department—many household gods to be destroyed, many errors to be corrected. This has been well illustrated in the case of Dr. Leuf, of Philadelphia, whose articles in the *Medical and Surgical Reporter* have been quite a revelation to many. The doctor has ably shown that many popular works on this subject are, to say the least, fallacious in their teachings and erroneous in their deductions. As an example of erroneous teaching, we have only to take up the subject of so-called abdominal breathing. This method is claimed by many excellent teachers of music to be the only true one, and that any other will surely lead the victim into consumption or else destroy the voice in time. If any one will take the trouble to investigate for himself or to read his works on anatomy, he will be quickly convinced that forcible expansion of the abdominal walls must lead, in the end, to injury. What should be aimed at, in the art of breathing, is a flexible condition of the thorax; and the muscle which will bring about this flexibility, principally, is the serratus magnus. The diaphragm will take care of itself; for if the ribs are well drawn out by the serrati muscles, it will quickly descend, without any effort on the part of the subject.

Another point, which has been overlooked, is what we will term: individualizing the person—a method always adopted by the successful physician when engaged in diagnosticating disease. By this method the strong points of the person in question can be ascertained and the weak points developed. Most men are weak in the abdominal region, and for that reason this should be the part first attended to. And, moreover, in my opinion, it is the most important part of the body to be developed, for various reasons best known to physicians. The muscles of this part of the body, together with the serrati muscles, should be plainly visible to the naked eye; and when a man is thus developed, the developing of the rest of the body is accomplished with comparative ease. The main object should be the uniform development of the whole body, never exercising one or two parts at the expense of the others. This point can be readily proven by a visit to one of the German "Turn-Schools," or—as we call them—gymnasiums. There one can see pupils who are experts in certain feats, but whose general make-up is not flattering to the system.

When a man is once properly developed, he will remain so, provided, he practises a correct carriage of body and a proper method of breathing. This is well illustrated in the person of a certain Mr. Checkly, of Brooklyn, who is at present engaged in teaching physical culture. This man claims to have taken no special exercise previous to last January, for the space of ten years; yet at the end of that time he was able to handle his body with far greater ease than many men who were engaged constantly in gymnasiums.

What we need in this country are institutions devoted to rational gymnastics, also to the study of physiology, anatomy, hygiene and instruction in the art of cooking; for it must be remembered that the ordinary trainer knows what kind of food is necessary for his pupil. This instruction is vital; for without the proper fuel, no matter how good the machinery, we cannot get the same results. If a man, or woman, could show a diploma from an institution conducted on these principles, then we might expect to see men and women who would not only be a credit to themselves but a great gain to the state. As a result of such teachings men would have more regard for their bodies and be less likely to abuse them by vile practices; and society in general would put cn a different aspect. Instead of crowding rum-shops and other places of evil resort, they would flock to the gymnasiums or the open fields, there to exercise their new found powers, which, instead of making them feel wearied or exhausted, would, on the contrary, better prepare them for the succesful prosecution of their business.

It remains with the medical profession especially, to bring about this grand result, and also to interest philanthropists in a scheme that will be far more beneficial to humanity than the erection of a thousand hospitals.

